# Assessment of Microbial Community Structure and Function in Serially Passaged Wastewater Electro-Bioreactor Sludge: An Approach to Enhance Sludge Settleability

**DOI:** 10.1038/s41598-018-25509-2

**Published:** 2018-05-03

**Authors:** Nancy A. ElNaker, Maria Elektorowicz, Vincenzo Naddeo, Shadi W. Hasan, Ahmed F. Yousef

**Affiliations:** 1 Department of Chemistry, Khalifa University of Science and Technology, Masdar City Campus, PO Box, 54224 Abu Dhabi, United Arab Emirates; 2 Department of Chemical Engineering, Khalifa University of Science and Technology, Masdar City Campus, PO Box, 54224 Abu Dhabi, United Arab Emirates; 3 Department of Building, Civil and Environmental Engineering, Concordia University, 1455 Blvd de Maisonneuve W., Montreal, Quebec, H3G 1M8 Canada; 4 Department of Civil Engineering, University of Salerno - Via Giovanni Paolo II #132, 84084 Fisciano (SA), Italy

## Abstract

Several studies have been carried out to understand bulking phenomena and the importance of environmental factors on sludge settling characteristics. The main objective of this study was to carry out functional characterization of microbial community structure of wastewater electro-bioreactor sludge as it undergoes serial passaging in the presence or absence of a current density over 15 days. Illumina MiSeq sequencing and QIIME were used to assess sludge microbial community shifts over time. (α) and (β) diversity analysis were conducted to assess the microbial diversity in electro-bioreactors. A phylogeny-based weighted UniFrac distance analysis was used to compare between bacterial communities while BIO-ENV trend and Spearman’s rank correlation analysis were performed to investigate how reactor operational parameters correlated with bacterial community diversity. Results showed that the removal efficiency of soluble chemical oxygen demand (sCOD) ranged from 91–97%, while phosphorous (PO_4_^3−^-P) removal was approximately 99%. Phylogenetic analysis revealed stark differences in the development of sludge microbial communities in the control and treatment reactor. There was no mention of any studies aimed at characterizing functional microbial communities under electric field and the results communicated here are the first, to our knowledge, that address this gap in the literature.

## Introduction

In recent years, several advances have been made to optimize wastewater treatment plants design and operation. The quality of effluent is dependent on the bioreactor’s microbial community structure and dynamics as well as the method by which the biological sludge mass can be separated from the treated wastewater. In activated sludge process, the solids/liquid separation typically takes place through a separation of spontaneously aggregated flocs by gravity sedimentation in secondary clarifiers^1^, while in membrane bioreactors, the biosolids are separated by means of a polymeric membrane based on microfiltration or ultrafiltration^2^. The integration of electrochemical processes into wastewater treatment bioreactors combine biodegradation, electrochemical, and sludge separation processes into one system, achieving high effluent quality when compared to conventional process^3–5^. Current density (CD) is a crucial factor which affects the performance of electrochemical treatment in electrically enhanced wastewater treatment bioreactors, controlling the dosing rate of metal ions and the gas bubble density that are released in the solution via redox reactions of electrodes^6^. The use of sacrificial anodes releases metal ions which precipitate or adsorb negatively charged foulants such as extracellular polymeric substances and soluble microbial products, and facilitates the formation of larger flocs which drives the particle back transport from the membrane surface to the bulk solution^7^. In addition to these physical effects, biological effects of incorporating electrical current into wastewater treatment reactors have also been reported. Electrochemical stimulation could stimulate the biodegradation of organic contaminants by providing electron acceptors and donors for microorganisms performing these reactions^8^. Furthermore, the application of electrical current in anaerobic wastewater treatment systems can stimulate organic biodegradation and reactor stability which is associated with enrichment of certain microbial functional species in biofilm that formed on the both the anode and cathode^9,10^.

Several studies have been carried out to understand bulking phenomena and the importance of environmental factors on sludge settling characteristics, which are thought to be strongly influenced by flocculation, sludge bulking, foaming and rising^2^. Wastewater treatment plants’ bacterial community structure is now becoming the focus of bioreactor operation monitoring to avoid many technical problems. Filamentous bacteria are a necessary component of the activated sludge, but their excessive growth results in sludge bulking and foaming, two of the most common technical problems of wastewater treatment plant bioreactor operation^11–13^. Based on 16S rRNA gene sequencing, bulking and foaming bacteria^14^ and functional interactions between various groups of bacteria that perform nitrogen fixation, nitrification, ammonification, and other biochemical processes have been shown for an entire wastewater treatment plant bioreactor, and the temporal dynamics of bacterial communities have also been studied^15,16^.

High-throughput sequencing technologies have significantly improved researchers’ ability to investigate microbial communities in various municipal and industrial wastewater treatment plant^12,17,18^. Indeed, Illumina MiSeq has been successfully used to study various environmental and industrial systems in recent years^19^. In this study, activated sludge samples were collected from the membrane bioreactor plant at Masdar city and were analyzed using Illumina MiSeq after being used as inoculum in serially passaged batch electro-bioreactors operated at CD of 3 and 7 Am^−2^ for 15 days. The sequencing data was analyzed using Quantitative Insights Into Microbial Ecology (QIIME^TM^) in order to elucidate alpha (α) and beta (β) diversity present in the different test reactors. Our data is the first to investigate the impact of serial passaging on the performance and microbial community structure of wastewater electro-bioreactors. The main objectives of this study were (a) to investigate the effect of CD on the performance of serially passaged electro-bioreactors; (b) to illustrate the effect of serial passaging on microbial community structure and function; (c) to differentiate between the effects of CD and serial passaging linking the performance and functional bacterial groups associated with sludge settling problems in both bioreactors and electro-bioreactors. This research is the first to study the integration of serial passaging technique with bio-electrochemical stimulation on the microbial communities and reactor performance efficiency and stability in wastewater electro-bioreactors.

## Results and Discussion

### *sCOD* and phosphorus removal in serially passaged wastewater reactors

**Figure 1:**
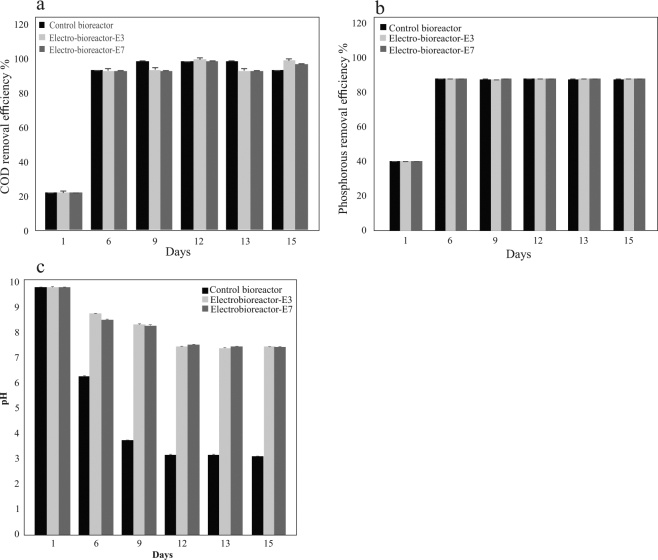
Removal efficiencies of (**a**) COD, (**b**) PO_4_^3−^-P in serially passaged control bioreactor and electro-bioreactors E3 and E7 operated at current densities 3 and 7 Am^−2^ respectively. Overall, the removal efficiency of sCOD and PO_4_^3−^-P in electro-bioreactors is attributed to the effects of electrochemical processes in addition to biodegradation. (**c**) pH variations in serially passaged control bioreactor and electro-bioreactors E3 and E7 operated at current densities 3 and 7 Am^−2^ respectively. Results confirmed that serially passaged electro-bioreactors maintained the pH which in turn stabilized the medium for the bacteria to be activated.

To assess the performance and efficiency of serially passaged wastewater bioreactors and electro-bioreactors, sCOD and PO_4_^3−^-P concentrations were measured in the effluent (Fig. 1a and b). As shown, sCOD removal efficiency ranged from 91–98% in serially passaged control bioreactors and electro-bioreactors operated at CD of 3 and 7 Am^−2^. It was observed that passaging days 9, 12 and 13 had the highest sCOD removal of 97% in control bioreactor then slightly decreased to 91% by day 15, while the highest sCOD removal was 97–98% in electro-bioreactors operated at CD of 3 and 7 Am^−2^ on days 12 and 15. The high removal efficiency of sCOD in control bioreactors is due to the short hydraulic control of sludge age in terms of organic loading to recycled sludge across the passaging days. However, the decline observed on day 15 could be due to the dispersion of the activated sludge flocs which causes turbidity resulting in high sCOD concentration in the effluent; these results are in agreement with previous findings which confirms that sludge settling is an important factor affecting organic removal^2^. Nutrient removal was also investigated by reporting the removal efficiency of PO_4_^3−^-P in all serially passaged wastewater reactors tested. PO_4_^3−^-P was efficiently removed reaching 99% in all reactors during the whole serial passaging period. The high phosphorus removal in the control bioreactor could be due to biodegradation process such as the presence of polyphosphate accumulating organisms (PAOs) which are a relatively fast growing heterotrophs which can remove PO_4_^3−^-P. Overall, the removal efficiency of sCOD and PO_4_^3−^-P in electro-bioreactors is attributed to the effects of electrochemical processes (i.e. electrocoagulation, electro-osmosis and electrophoresis) in addition to biodegradation which was previously explored by^4,20^. It should be noted that no significant evolution of applied voltage was reported. Results revealed an average of 5.1 ± 1.1 V/cm over 15 days of experimental investigations in the electro-bioreactors operated at CD of 3 and 7 Am^−2^.

### pH variations in serially passaged wastewater reactors

One of the physiochemical factors affecting the bioreactors and electro-bioreactors performance is the pH of the solution^21^. Also, studies on activated sludge systems show that most of biological systems and bacteria are activate in pH ranges of 4 to 9^22^. As shown in Fig. 1c, pH was initially 9.2 ± 0.2 in all reactors tested during the serial passaging period. In control bioreactor pH declined by 36% to 5.9 ± 0.2 after 5 days of passaging then decreased to 3.5 ± 0.1 by day 9 then stabilized at 2.9 ± 0.1 from day 12 to 15 end of serial passaging. In electro-bioreactors operated at CDs of 3 and 7 Am^−2^, pH decreased by 11–14% to 8.2 ± 0.3 and 7.9 ± 0.2 after 5 days of passaging then by approximately 6% to 7.8 ± 0.1 by day 9 then stabilized in the range (6.9 ± 0.1–7.0 ± 0.1) from day 12 to 15 at CDs of 3 and 7 Am^−2^. Therefore, pH of the solution in control bioreactors and electro-bioreactors declined by 69 and 23%, respectively. This means that serially passaged electro-bioreactors maintained the pH which in turn stabilized the medium for the bacteria to be activated. Additionally, this confirms the high removal efficiency of sCOD and PO_4_^3−^-P as previously reported that depending on the pH of the solution, various metal species are produced during electrocoagulation which react with the pollutants leading to the destabilization and aggregation of suspended particles and the precipitation and adsorption of dissolved contaminants^21^. Studies aimed at understanding bacterial community flocculation determined that one aspect of flocculation is dictated by ionic interactions between organic polymers that are secreted into the environment by various bacteria. These organic polymers, mostly made up of protein and carbohydrates, vary in size and this is affected by enzymatic activity^23^. It is widely accepted that pH affects enzymatic activity, therefore fluctuations in pH can result in different organic polymer lengths and ionic charges, and ultimately affects biological flocculation. This change in pH could be one reason why the activated sludge particles in the control bioreactor did not settle and were kept in suspension.

### Sludge volume index (SVI) and settling velocity (Vo) variations in serially passaged wastewater reactors

Sludge volume index (SVI) is often used to characterize the settleability of a specific sludge in treatment plants. It is the volume in milliliters occupied by 1 g of a suspension after 30 min settling. SVI was measured at the end of each experiment after 15 days in serially passaged control bioreactor and electro-bioreactors operated at CD of 3 and 7 Am^−2^. Results showed that SVI was reduced from 325 to 236.6, 141.5, and 54.7 mLg^−1^ in serially passaged control bioreactor and electro-bioreactors operated at CD of 3 and 7 Am^−2^, respectively reflecting the importance of applying electric field in the sludge cohesion and density. Sludge settling velocity (*V*_*o*_) was calculated based on the mathematical formula by Akca *et al*.^24^:

1 $${V}_{o}=28.1\,{(SVI)}^{-0.2667}$$

according to the aforementioned formula, *V*_*o*_ of 6, 6.5, 7.5 and 9.7 mh^−1^ was reported in the control bioreactor, and electro-bioreactors operated at CD of 3 and 7 Am^−2^, respectively. Those results show a significant improvement on the settling velocity (up to 161.7%) via creating denser flocs which tend to settle at a faster rate.

### Effect of serial passaging on microbial diversity in wastewater bioreactors

**Figure 2:**
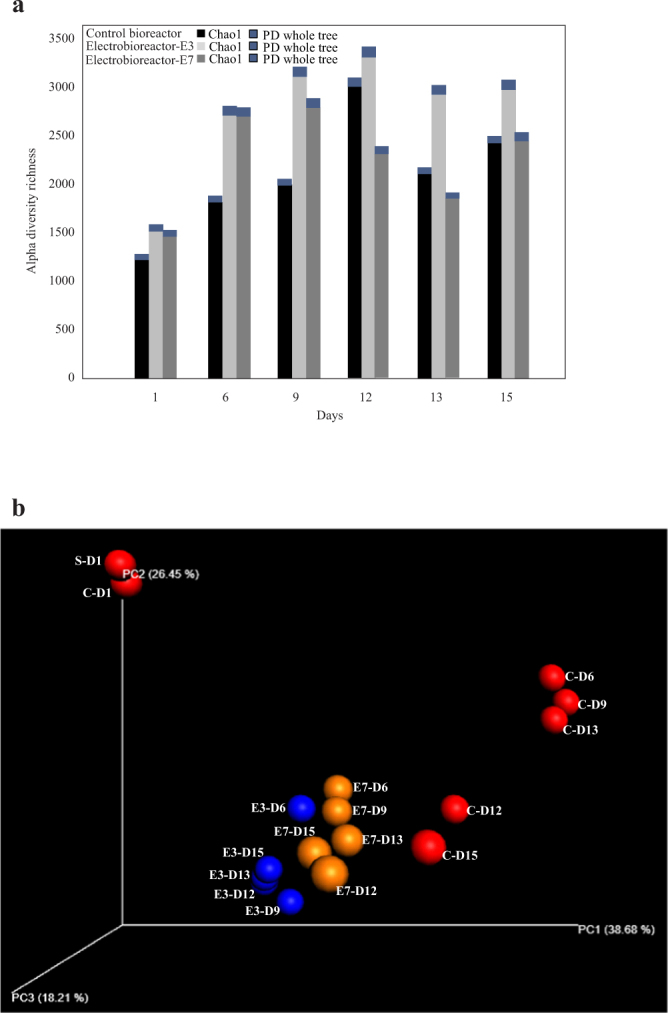
(**a**) Alpha diversity richness calculations using Chao1 and PD whole tree methods, (**b**) three-dimensional principal coordinate analysis (PCoA) plot showing the bacterial community variations present in serially passaged control bioreactors (red data points) and electro-bioreactors E3 (blue data points) and E7 (orange data points). It is noted that bacterial communities in electro-bioreactors clustered together when compared to control bioreactor. (S-D1: Fresh sludge sample without feeding with synthetic wastewater; C-Di: Control sample in day i; E-Dj: Electro-bioreactor sample in day j).

To assess the microbial diversity in the serially passaged bioreactor and electro-bioreactors subjected to CDs of 3 and 7 Am^−2^, we performed alpha (α) diversity analysis on each sample and beta (β) diversity analysis across the samples. α diversity expresses the diversity of a population within a system; a community will have a higher α diversity when there is a higher number of unrelated species within the same sample^25^. α diversity was assessed by using the *Chao1* index and Phylogenetic Diversity (PD) whole tree index^26^. The results of these measurements indicated differences in α diversity depending on passaging days. Specifically, control bioreactors and electro-bioreactors operated at 3 Am^−2^ started with low α diversity and continued to increase reaching the highest richness (peak) by day 12 (Fig. 2a). α diversity then lowered by day 13 and 15 in both control and electro-bioreactors as shown in Fig. 2a. Additionally, communities present in electro-bioreactors operated at 7 Am^−2^ had the highest α diversity by day 9 then lowered by day 12, 13 and 15 as shown in Fig. 2a. Furthermore, control bioreactors and electro-bioreactors operated at CD of 3 Am^−2^ had a higher α diversity than electro-bioreactors operated at 7 Am^−2^.

To more clearly compare the bacterial communities, a phylogeny-based weighted UniFrac distance analysis was used^16^. The bacterial communities prior to applying electric field taking into consideration the serial passaging time effect were highly distinct from each other as shown in the Principle Coordinate Analysis (PCoA) plot (Fig. 2b). As can be seen from the PCoA plot, fresh sludge sample without feeding with synthetic wastewater (S-D1) and control bioreactor sample fed with synthetic wastewater (C-D1) clustered together and were very distant from the rest of the reactors tested. Electro-bioreactors operated at CD of 3 (blue data points) and 7 Am^−2^ (orange data points) clustered together, indicating that the bacterial communities that developed in those reactors were more similar than any of the control bioreactors. Control bioreactor samples at days 6, 9 and 13 (C-D6, C-D9 and C-D13) clustered together on the extreme right side of the plot, while control bioreactors at days 12 and 15 (C-D12 and C-D15) clustered together in closer proximity to the electro-bioreactor samples. Taken together, this data indicates that applying an electric current differentiated the microbial community present in the electro-bioreactors and this caused early stabilization in the community since all data points clustered together. In contrast, the bacterial community in the control bioreactors seemed to fluctuate as the sludge was serially passaged.

A BIO-ENV trend and Spearman’s rank correlation analysis (using QIIME v 1.9.0) was performed (β-diversity) to investigate for electric field and operational parameters that is correlated with bacterial community diversity. This type of analysis calculates a bacterial community difference distance matrix and compares to Euclidian distance matrixes for each of the other measured variables (such as temperature, pH, ammonia content etc.). The variables that best explained the weighted Unifrac distance between all samples were best correlated with incorporation of current density, PO_4_^3−^-P, sCOD and pH (correlation = 0.412, 0.197, 0.111 and 0.496, respectively).

### Clustering and phylogenetic analysis of microbial community structure

Raw Illumina MiSeq sequencing data was analyzed using QIIME for the 12 serially passaged reactor samples discussed here. Assigning sequences to different OTUs resulted in a total of 7019 OTUs with a minimum sequence depth of 24647 per sample and a table density of 0.404. Furthermore, after trimming and filtering according to the criteria mentioned in the methods section, the OTU biom table resulted in 6863 observed OTUs with a minimum sequencing depth of 24567. Rarefaction analysis of our sequencing data indicated that the sequencing depth we achieved per sample was sufficient to uncover low abundance species as rarefaction plots of reads vs OTU’s leveled off.

**Figure 3:**
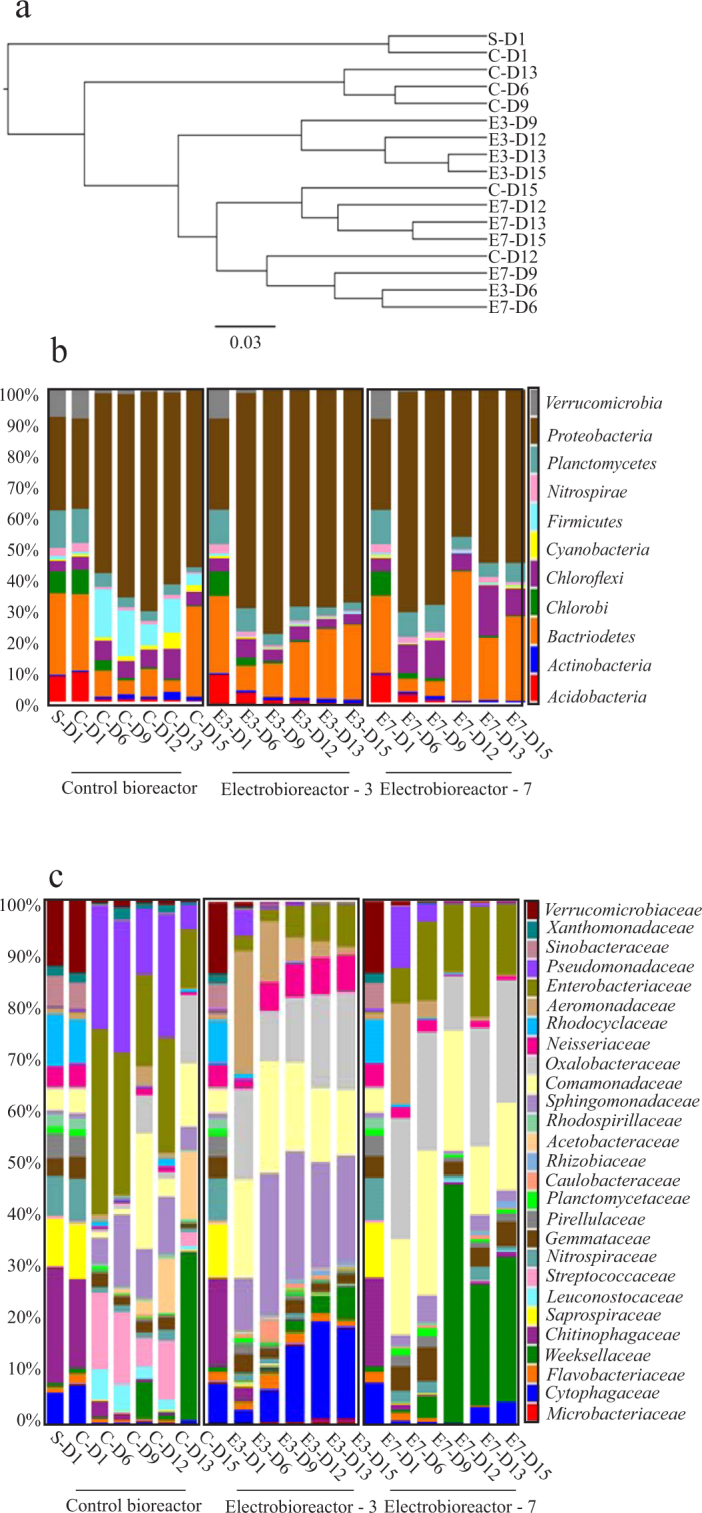
(**a**) Beta-diversity using UPGMA clustering analysis using weighted Unifrac. Phylogeny of the bacterial communities in the control bioreactor and electro-bioreactors at (**b**) phylum level, and (**c**) family level. It is concluded that serial passaged reactors had an impact on microbial community structure and composition in the presence and absence of electric field. (S-D1: Fresh sludge sample without feeding with synthetic wastewater; C-Di: Control sample in day i; E-Dj: Electro-bioreactor sample in day j).

β-diversity analysis using UPGMA clustering revealed that the bacterial communities in the 12 samples could be clustered into two main groups containing: (i) fresh sludge sample and control bioreactor containing sludge fed with synthetic wastewater on day 1. (ii) serially passaged control bioreactors (C-D6 to D15) and electro-bioreactors operated at CD of 3 (E3-D6 to D15) and 7 Am^−2^ (E7-D6 to D15) (Fig. 3a). The second main branch is divided into 2 sub-branches according to the absence and presence of electric current. As can be seen in Fig. 3a, bacterial communities in control bioreactors clustered together on days 6, 9 and 13 except from days 12 and 15 which clustered with communities from the electro-bioreactor operated at CD of 7 Am^−2^. Whereas microbial communities from the electro-bioreactors operated at 3 Am^−2^ clustered together on all passaging days except from day 6 which clustered with communities from the electro-bioreactor operated at 7 Am^−2^ from the same day. Additionally, communities from the serially passaged electro-bioreactor operated at 7 Am^−2^ were separated because of serial passaging. For example, communities from days 6, 9 and 12 clustered together on one branch separated from another branch containing communities from days 12, 13 and 15. This means that the serial passaged reactors had an impact on microbial community structure and composition in the presence and absence of electric field. That could be explained referring to the consequences of serial passage in terms of evolutionary impact of environment on passaged microorganisms. In other words, the adaptation of microorganisms to the utilization of new substrates. An ability to utilize a new substrate has the potential to increase growth rates as well as organism end point-densities^27^.

Phylogenetic analysis of the microbial communities present in the serially passaged reactors showed that *Proteobacteria* accounted for the largest fraction (59.7% average abundance across all samples) which is consistent with previous work^28,29^, followed by *Bacteroidetes* (16.7%), *Chloroflexi* (6.5%), *Planctomycetes* (5.3%), *Firmicutes* (3.3%), *Acidobacteria* (1.9%), *Chlorobi* (1.7%), *Verrucomicrobia* (1.6%) and *Nitrospirae* (1.2%) (Fig. 3b). In municipal WWTPs, the phylum *Proteobacteria* predominates, of which *Betaproteobacteria* (25.2% average abundance across all samples) is the most abundant class, largely responsible for organic and nutrient removal. The *Proteobacteria* encompass enormous morphological, physiological and metabolic diversity, and are of great importance to global carbon, nitrogen and sulphur cycles^11^. The results showed that the highest relative abundance of *Proteobacteria* members was in the serially passaged electro-bioreactor operated at CD of 3 Am^−2^ (63.3%) higher than the control bioreactor and electro-bioreactor operated at CD of 7 Am^−2^ (56.7 and 53.9%, respectively). A sub-dominant phyla *Bacteroidetes* correlated with serially passaged electro-bioreactor operated at CD of 7 Am^−2^ comprising a relative abundance of (20.2%) slightly higher than that of CD 3 Am^−2^ and control bioreactor (17.7 and 13.1%, respectively). This means that the application of electric field could have a positive effect on serially passaged microbial communities in wastewater reactors. It is noticed that when comparing the relative abundances of bacterial communities present in serially passaged electro-bioreactors and control bioreactor, there was an inflection point at certain passaging days where it reached its highest abundance then slightly decreased which could be due to serial passaging effect. For example, the relative abundance of *Proteobacteria* had the largest abundance on day 12 (70.4%) in control bioreactor, day 9 (77.2%) in electro-bioreactor operated at CD of 3 Am^−2^ and day 6 (70%) in electro-bioreactor operated at CD of 7 Am^−2^. Additionally, the average relative abundances of *Chloroflexi* in serially passaged electro-bioreactor operated at 7 Am^−2^ was higher than in control bioreactor and very low abundance in electro-bioreactor at 3 Am^−2^ (9.2, 5.9 and 0.4%, respectively). Previous studies reported that *Chloroflexi* as predominating filaments in municipal and domestic wastewater treatment plants^30,31^. Identification of filaments responsible for bulking enables to select optimal solution for their removal from a particular wastewater treatment plants. Furthermore, members of *Firmicutes phyla* known to occur in wastewater treatment plants treating penicillin-containing wastewater had the highest relative abundance in serially passaged control bioreactors and was depleted in both electro-bioreactors of CD 3 and 7 Am^−2^ with average relative abundance (8.6, 0.4 and 0.5%, respectively).

To more closely analyze the microbial community abundance and dynamics in the serially passaged reactors, a phylogenetic analysis at family level was constructed. After trimming and filtration, there are 27 families present with higher than 0.3% average abundance across all samples as shown in (Fig. 3c). *Comamonadaceae* (14.1%), *Enterobacteriaceae* (13.1%), *Oxalobacteraceae* (12.7%), *Sphingomonadaceae* (10%), *weeksellaceae* (9.7%), *Pseudomonadaceae* (7%), *Cytophagaceae* (5%) and *Aeromonadaceae* (4.8%) were the top abundant families across all samples. Our results showed that *Enterobacteriaceae*, *Pseudomonadaceae*, *Streptococcaceae*, *Acetobacteraceae* and *Rhodocyclaceae* had the highest average relative abundance across serially passaged control bioreactor samples than electro-bioreactors operated at CD of 3 and 7 Am^−2^ (18.8, 4.6 and 11.7%; 15, 1.2 and 2.7%; 8, 0.1 and 0.1%; 4.5, 0 and 0%; 2.1, 1.6 and 1.5%, respectively). This explains the high removal of sCOD and PO_4_^3−^-P (Fig. 2a,b) in control bioreactors on passaging days (6, 9 and 12) due to the high relative abundance of heterotrophic bacteria affiliated with organic and nutrient removal on these days such as *Pseudomonadaceae*; hydrocarbon utilizing organisms^32^, *Streptococcaceae*; glucose fermenting bacteria^33^ and *Rhodocyclaceae*; Phosphate accumulating organisms PAOs^34^. These families survived over time in serially passaged control bioreactors compared to both electro-bioreactors. *Sphingomonadaceae* (known to metabolize and remove micro-pollutants, such as polycyclic aromatic hydrocarbons (PAHs) and bisphenol A (BPA) degradation from wastewater^35^, Cytophagaceae; are Epiphetic protein hydrolyzers^36^, *Aeromonadaceae*; has been explored previously to treat sewage containing pharmaceuticals and personal care products (PPCPs)^37^, *Neisseriaceae*; involved in sCOD reduction in dairy wastewater^38^ and *Flavobacteriaceae*; heterotrophic bacteria associated with nutrient and micro-pollutant removal^39^ had the highest average relative abundance in serially passaged electro-bioreactors operated at CD of 3 Am^−2^ than in control bioreactor and electro-bioreactors operated at 7 Am^−2^ (17.3, 7.4 and 2.3%; 11.3, 1.9 and 2.8%; 7.6, 1.4 and 4.2%; 5.4, 1.4 and 1.8%; 2, 0.8 and 0.8%, respectively). These families continued to increase and survived through the serial passaging in the electro-bioreactor operated at CD of 3 Am^−2^ except for *Flavobacteriaceae* which started to decrease in abundance after day 9. *Oxalobacteraceae*; members of the family are heterotrophic and some genera fix nitrogen which are applied as plant growth-promoting bacterial inoculants in agriculture^40^, *Comamonadaceae*; is reported to be capable of performing a biological nutrient removal process with a reduced increase in cell mass when the oxygen supply is limited “see Table 1”^41^ and *Weeksellaceae*; present in hydrocarbon-polluted soil microbial communities or natural asphalts^32^ had the highest average relative abundance in serially passaged electro-bioreactors operated at CD of 7 Am^−2^ than in control bioreactor and electro-bioreactors operated at 3 Am^−2^ (17.2, 4.0 and 12.8%; 17.1, 7.3 and 14.6%; 17, 6.9 and 2.4%, respectively).

**Table 1:** OTU counts of bacterial families in serially passaged control bioreactor.

Bacterial families	Day 1	Day 6	Day 9	Day 12	Day 13	Day 15
*Comamonadaceae*	1956	53,641	64,816	79,380	49,229	43,552
*Flavobacteriaceae*	988	801	607	505	295	82
*Verrucomicrobiaceae*	6357	411	1432	700	781	179
*Pseudomonadaceae*	9630	20,479	31,581	16,998	22,925	5463
*Neisseriaceae*	1986	339	584	1952	833	586
*Enterobacteriaceae*	193	29,420	29,259	20,948	19,123	11,366
*Streptococcaceae*	201	12,154	15,042	6560	9907	2701

### Linking functional microbial community function to sludge settling in serially passaged reactors

Bacterial communities that are responsible for activated sludge bulking and foaming play a role in the degradation of substrates such as fatty acids, amino acids and other bioorganic compounds^2^. These are *Comamonadaceae, Pseudomonadaceae*, *Verrucomicrobiaceae* and *Flavobacteriaceae*. These families are the major components of activated sludge of most wastewater treatment plants worldwide, playing crucial roles in degradation of organic compounds and forming floc structure of activated sludge. Moreover, many inductors of activated sludge bulking and foaming efficiently degrade stable organic compounds and their increase in content and biodiversity is reasonable^42–45^. *Filamentous* bacteria are usually present in wastewater treatment plants in a low number supporting the formation of microbial structures. The excessive growth of these bacteria causes sludge bulking^11^.

**Figure 4:**
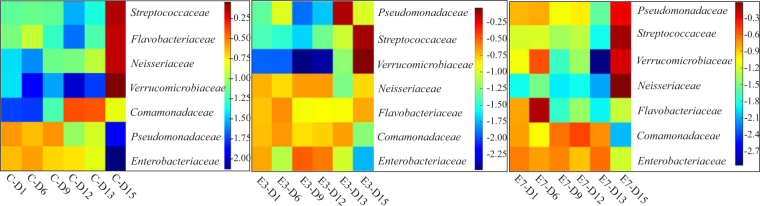
Heat map of functional bacterial families in serially passaged control bioreactor and electro-bioreactors E3 and E7. It was observed that microbial subpopulations associated with sludge bulking and foaming are different in electro-bioreactors when compared to control bioreactor. (C-Di: Control sample in day i; E-Dj: Electro-bioreactor sample in day j).

Therefore, it is important to assess the microbial subpopulations of functionally important bacteria in our serially passaged reactors, a heat map was constructed illustrating these differences (Fig. 4). This heat map, which is only based on functionally interesting bacterial families affiliated with sludge bulking and foaming (*Comamonadaceae*, *Flavobacteriaceae*, *Verrucomicrobiaceaea* and *Pseudomonadaceae*) and pathogenic microflora (*Neisseriaceae*, *Enterobacteriaceae* and *Streptococcaceae*)^46^, clearly differentiates the behavior of these bacteria when it is serially passaged in control bioreactor and electro-bioreactors. In serially passaged control bioreactors, the abundance of *Comamonadaceae* increased reaching the maximum OTU count by day 12 then slightly decreased and stabilized (see Table 1). The behavior was similar for *Pseudomonadaceae*, *Streptococcaceae* and *Enterobacteriaceae* which tend to an initial increase with highest abundance by day 9. *Enterobacteriaceae* was high in abundance till day 6 then continued to slowly decrease till the end of serial passaging while *Pseudomonadaceae* and *Streptococcaceae* dramatically decreased by day 12 then slightly increased on day 13 then decreased again by day 15. *Neisseriaceae* and *Verrucomicrobiaceae* members initially decreased then reached the maximum OTU count by day 12 then decreased and stabilized by day 15. *Flavobacteriaceae* had an inverse correlation by days as it tends to decrease from day 1 to day 15 which means that this bacterial family could not survive and depleted in the serially passaged control bioreactor.

In serially passaged electro-bioreactors operated at CD of 3 and 7 Am^−2^ (Tables 2 and 3), *Comamonadaceae* and *Flavobacteriaceae* was initially increased in abundance with highest OTU counts by day 9 at CD of 3 Am^−2^ and day 12 at CD of 7 Am^−2^ then deceased and stabilized. *Verrucomicrobiaceae* and *Streptococcaceae* were depleted through the 15-day while *Pseudomonadaceae* had the highest OTU count by day 6 then depleted by the end of serial passaging in both electro-bioreactors operated at 3 and 7 Am^−2^. Additionally, *Enterobacteriaceae* were in low abundance till day 9 then started to occur with high OTU counts by day 12 till day 15 of the serial passaging. Bacterial family *Neisseriaceae* had a different behavior through the serial passaging days which tended to increase in abundance through the 15-day of serial passaging in electro-bioreactor operated at CD of 3 Am^−2^ while dramatically decreased by day 12 in electro-bioreactor operated at CD of 7 Am^−2^.

**Table 2:** OTU counts of bacterial families in serially passaged electro-bioreactor operated at CD of 3 Am^−2^.

Bacterial families	Day 1	Day 6	Day 9	Day 12	Day 13	Day 15
*Comamonadaceae*	1956	18,040	22,959	21,225	13,443	16,059
*Flavobacteriaceae*	988	1476	3527	2897	1482	1828
*Verrucomicrobiaceae*	6357	1077	243	279	69	76
*Pseudomonadaceae*	275	4847	862	423	132	107
*Neisseriaceae*	1986	1706	5872	8429	6890	9069
*Enterobacteriaceae*	193	2565	2579	7650	7086	10,874
*Streptococcaceae*	201	60	26	35	32	18

**Table 3:** OTU counts of bacterial families in serially passaged electro-bioreactor operated at CD of 7 Am^−2^.

Bacterial families	Day 1	Day 6	Day 9	Day 12	Day 13	Day 15
*Comamonadaceae*	1956	17,636	25,367	26,504	2261	15,370
*Flavobacteriaceae*	988	1649	538	7	37	79
*Verrucomicrobiaceae*	6357	696	357	188	50	207
*Pseudomonadaceae*	275	11,301	2953	219	101	210
*Neisseriaceae*	1986	2041	2117	357	200	784
*Enterobacteriaceae*	193	6658	13,913	15,035	3712	12,404
*Streptococcaceae*	201	56	62	12	7	26

Our results revealed that microbial communities structure changed in serially passaged sludge in all reactors tested. This could be due to the two phenomena involved in serial passaging experiments which are population growth of the microorganisms and the movement that occurs during passage (i.e., the dilution/transmission step). There can be differences in both the quality and intensity of selection imposed at each of these steps taking into consideration the duration of growth in the course of a single passage (difference between passaging days)^28,47^. Moreover, microbial communities structure, diversity and response changed in the serially passaged reactors which could be beneficial by optimizing the operating conditions and adjusting the duration and gap between the passaging days in bioreactor and electro-bioreactors treating wastewater.

This study indicates that the introduction of serial passaging, as a new approach to electro-bioreactors treating wastewater, is associated with greater sludge bulking and microbial community stability. Indeed, this approach represents a strategy that can be employed to solve sludge bulking and foaming problems, leading to improvement in sludge settleability and a more efficient waste water treatment process. The results reported here are the first to describe the effects of current density on microbial community structures in serially passaged sludge.

## Methods

### Electro-bioreactor experimental design

**Figure 5:**
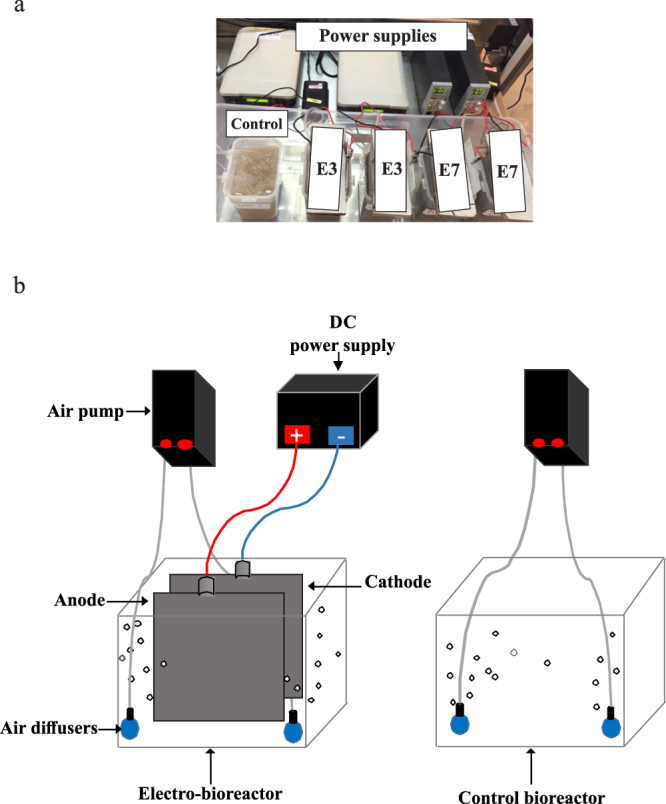
(**a**) Photograph and (**b**) schematic diagram of the experimental setup of serially passaged control bioreactor and electro-bioreactors operated at current densities 3 and 7 Am^−2^.

The experiments in this research study were conducted to evaluate the microbial community under different current densities of 3 and 7 Am^−2^ using a hydraulic retention time of 6 h. The purpose of this design was to determine the operating conditions that favor an optimal effluent quality and microbial community. The selected range of applied CDs was according to a previous study by our group^48^ and industrial scale membrane bioreactor wastewater treatment plants^49^. All bioreactors batch were fed with synthetic wastewater (0.2% Glucose, 1.5 mM ammonium sulfate, 270 μM potassium phosphate, 160 μM magnesium sulfate, 20 μM manganese sulfate, 1.47 μM iron (III) chloride, 20 μM calcium chloride, 330 μM potassium chloride, 300 μM sodium bicarbonate). Fresh activated sludge was collected from Masdar city’s membrane bioreactor wastewater treatment plant (Abu Dhabi – UAE) and used immediately to avoid any changes in its physiochemical and microbiological characteristics. The system used for this study was aerobic batch electro-bioreactors containing sludge and synthetic wastewater prepared in the laboratory. The total effective volume of the reactor is 1500 mL of which 300 mL of raw sludge was mixed with 1200 mL of synthetic wastewater. Four batch electro-bioreactors were connected to DC power supplies by immersing a pair of electrodes in each reactor as shown in Fig. 5. The sludge was serially passaged and fed with synthetic wastewater every 24 h for a two-week experiment. The sludge samples were collected then centrifuged and the supernatant was discarded. The sludge was then fed with synthetic wastewater and left to operate for 24 h and so on. Sampling was carried out on days 1, 6, 9, 12, 13 and 15. The electrodes used in all experiments consisted of rectangular sheets of perforated aluminum with 75% opening as the anode, and stainless steel as the cathode spaced 5 cm apart. The effective surface area of the anode was calculated in each operating condition depending on the effective volume of the sample, by multiplying the width by the immersed length of the anode in the bioreactor. Aeration was provided via small ceramic ball air stone diffusers (2-inch diameter), connected to air pumps placed at the bottom to provide oxygen necessary (>2 mgL^−1^) for aerobic microbial growth and sludge mixing. A reference control bioreactor (0 Am^−2^) was used in all conditions and had no electrodes.

### Synthetic wastewater and sludge characteristics

Synthetic wastewater and sludge dissolved oxygen (DO in mgL^−1^), pH, temperature (T in °C) and electrical conductivity (EC in μScm^−1^) were analyzed using a HACH HQ40d single-input multi-meter probe (Hach Company, Loveland, CO, USA). A 50-mL sample was collected from each reactor cell and centrifuged for 15 min at 3885 × g. The supernatant was analyzed by measuring soluble chemical oxygen demand (sCOD), phosphorous (PO_4_^3−^-P), ammonium (NH_4_^+^-N) and nitrate (NO_3_^−^-N) using HACH vials LCK 314–1014, LCK 348–349, LCK 303–304 and LCK 339–340, respectively according to manufacturer’s instructions. The oxygen uptake rate (OUR) was measured after cutting off aeration in all bioreactors. The DO probe (Hach HQ40d) was immersed in the sludge through which DO depletion was monitored by taking a reading every minute for 15 min. The slope of the DO vs time plot represents the OUR (in mgO_2_L^−1^ h^−1^). The current (I) supplied by the DC power supplies was determined based on the effective surface area (A_s_) of the anode and the desired operating current density (CD). Hydraulic retention time is the effective volume of aeration tank divided by the influent flowrate of wastewater.

### Microbial community analysis

Total genomic DNA was isolated from the samples collected from all the electro-bioreactors using the PowerSoil® DNA Isolation Kit (MOBIO Laboratories Inc. Carlsbad, CA, USA). The DNA samples were sent to Macrogen Inc. (Seoul, Republic of Korea) for Illumina MiSeq sequencing. Amplicon libraries were created using the 337F and 805R 16S V3-V4 universal primers (GACTCCTACGGGAGGCWGCAG and GACTACCAGGGTATCTAATC). The Illumina MiSeq instrument at Macrogen Inc. operates using control software v2.2 in conjunction with real time analysis software v1.18. Raw sequences delivered to our laboratory were analyzed by QIIME^TM^ (version 1.9.1) using published bioinformatics pipelines^50^. Before generating any figures, we filtered the QIIME^TM^ produced biom files by removing all unassigned operational taxonomic units (OTU’s) and any OTU that did not at least have 5 counts in at least one of the samples tested. (α) and beta (β) diversity analysis were conducted to assess the microbial diversity in the serially passaged bioreactor and electro-bioreactors^25^. A phylogeny-based weighted UniFrac distance analysis^16^ was used to compare between bacterial communities while BIO-ENV trend and Spearman’s rank correlation analysis was performed to investigate for electric field and operational parameters that is correlated with bacterial community diversity.

### Operational parameters

Sludge and synthetic wastewater influent and effluent operational parameters which are the dissolved oxygen DO, (mgL^−1^) and electrical conductivity EC, (μScm^−1^) were illustrated in (Table 4) showing 6 control bioreactor samples (C) and 6 electro-bioreactor samples (E) operated at current density of 3 Am^−2^ (i.e. E3) and 7 Am^−2^ (i.e. E7) serially passaged for 15 days.

**Table 4:** Operational parameters of serially passaged control bioreactor samples and electro-bioreactors samples E3 and E7.

Passaging days	Day 1	Day 6	Day 9	Day 12	Day 13	Day 15
C–DO, mgL^−1^	8.5	6.6	6.6	6.8	6.9	8.6
E3–DO, mgL^−1^	8.5	7.3	7.9	6.8	6.7	7.4
E7–DO, mgL^−1^	8.7	4.6	5.4	5	5.2	6.3
C–EC, μScm^−1^	486	567	299	745	891	902
E3–EC, μScm^−1^	369	113.6	100	88	87.2	97.7
E7–EC, μScm^−1^	385	161.4	123.9	114.9	113.8	104.9
